# Preventing, reducing, and attenuating restraint: A prospective controlled trial of the implementation of peer support in acute psychiatry

**DOI:** 10.3389/fpsyt.2023.1089484

**Published:** 2023-02-07

**Authors:** Julia Badouin, Andreas Bechdolf, Felix Bermpohl, Johanna Baumgardt, Stefan Weinmann

**Affiliations:** ^1^Department of Psychiatry, Psychotherapy and Psychosomatic Medicine, Vivantes Hospital Am Urban, Vivantes Hospital im Friedrichshain, Academic Hospital, Charité–University Medicine Berlin, Berlin, Germany; ^2^Department of Psychiatry and Neuroscience, Charité Campus Mitte Charité—University Medicine Berlin, Berlin, Germany; ^3^ORYGEN, National Centre of Excellence in Youth Mental Health, University of Melbourne, Melbourne, VIC, Australia; ^4^Wissenschaftliches Institut der AOK (WIdO), Berlin, Germany; ^5^Psychiatric Hospital, Theodor-Wenzel-Werk, Berlin, Germany; ^6^University Psychiatric Clinic (UPK), University Basel, Basel, Switzerland

**Keywords:** peer support, acute psychiatric care, predictor, recovery, restraint

## Abstract

**Introduction:**

The use of restraint as a means of managing patients is considered a critical factor that interferes with recovery. Strategies to create a less restrictive environment within psychiatric facilities are therefore eagerly sought. Peer support workers (PSWs) are increasingly employed in mental health settings. The prevailing theory is that PSWs have the potential to contribute to conflict and restraint prevention efforts in acute psychiatric wards. However, to date, research in support of this claim remains limited.

**Objective:**

The present study aimed at assessing the effectiveness of employing peer support workers with regard to reducing the use of restraint.

**Methods:**

This prospective controlled pre–post study sought to evaluate the implementation of peer support in one locked ward compared to treatment as usual (TAU) with no implementation of peer support in a second locked ward of a psychiatry department in Berlin, Germany. The pre–post comparison was planned to consist of two assessment periods of 3 months each, taking place directly before and after peer support implementation or TAU. Both assessments were extended to a period of 6 months, before and after the initially planned 12-month implementation process, in order to balance the effects of disruptions and of the COVID-19 pandemic. Using routine data, the proportion, frequency, and duration of mechanical restraint, forced medication as well as mechanical restraint in combination with forced medication, were evaluated.

**Results:**

In the control group, an increase in the proportion of patients subjected to measures of restraint was found between pre- and post-assessment, which was accompanied by a further increase in the mean number of events of restraint per patient within this group. In the intervention group, no significant change in the application of restraint was observed during the study period.

**Discussion:**

There is some indication that peer support may be protective with regard to restraint in acute wards. However, our study faced major challenges during the implementation process and the post-assessment period, such as COVID-19 and staff reorganization. This may have led to peer support not reaching its full potential. The relationship between the implementation of peer support and the use of restraint therefore merits further investigation.

## 1. Introduction

The United Nations Convention on the Rights of Persons with Disabilities emphasizes that the human rights of people subjected to different measures of restraint in psychiatric treatment settings are at considerable risk (1). There is a general consensus that restraint should only be considered as a last resort measure and governed by stringent regulations that clearly define when it may be applied (2–5). Reducing the use of restraint has become a priority in the mental health-care systems of many countries (2, 3). Though to date only partially realized, the development of preventive strategies is endorsed with virtual unanimity (6–9). Nevertheless, the complexity of the issue has posed a significant barrier to implementing such strategies (10).

Aiming to untangle this complexity by establishing evidence- and consensus-based standards, in 2018, the German evidence-based guideline “Prevention of Coercion: Prevention and Therapy of Aggressive Behavior in Adults” was introduced, soon followed by a 12-point program providing concrete implementation recommendations for psychiatric hospitals. Among other proposals designed to prevent the use of restraint in clinical practice, the program particularly highlights peer support as a promising, resource-oriented intervention (11, 12). While a considerable amount of research has been undertaken to evaluate interventions to reduce restraint in psychiatry, such as the Six Core Strategies (6SC) or the Safewards Model (13), the preventive effectiveness of peer support remains to be proven (11).

Within the psychiatric care system, acute wards face particular challenges due to the high density of people with acute psychiatric symptoms. Distress and despair experienced during a mental health crisis may lead to conflict, aggression, or violent behavior. Restraint is often used to contain situations evolving from these reactions (14, 15), even though its application may itself cause significant direct or indirect psychological or physical harm to patients and staff alike (16–18). In addition, measures of restraint are associated with a longer duration of inpatient treatment (19).

Rates of restraint vary across individual mental health units and across countries, with reliable transborder estimates being scarce. A German multicenter study found that 8% of psychiatric inpatients were exposed to restraint (20). The types of restraint measures vary between countries as well, depending on national psychiatric conventions and legislation (21–26). The use of seclusion and restraint was selected by the German Association for Psychiatry, Psychotherapy and Psychosomatics (DGPPN) as one of 10 parameters measuring the quality of mental health care for patients with schizophrenia (4), while further research has proposed it as an indicator for assessing longitudinal tendencies within medical facilities (5).

Peer support has been established as a key component of the empowerment and recovery movement, which endeavors to broaden the understanding of individual recovery (27, 28). In this conception, recovery can be captured in five major processes: the development of a sense of “connectedness,” “hope and optimism about the future,” “identity,” “meaning in life,” and “empowerment” (29). Thus, in contrast to a more conventional approach in psychiatry, with its focus on pathology and psychiatric symptoms, recovery constitutes a set of beliefs or a point of view centered around the greater participation and self-determination of people experiencing mental health crises (30). Current theories have built upon this understanding by repeatedly and plausibly hypothesizing that incorporating recovery-focused interventions into clinical practice effectively decreases patient aggression and the use of restraint measures (31–33).

Peer support workers (PSWs)—individuals with lived experiences of mental health challenges—impart personal experiential knowledge to people currently experiencing mental health crises, working from a foundation of reciprocal, symmetrical relationships (34–37). A previous study suggests that peer support operates through three primary mechanisms: (1) establishing bonds and a sense of alliance anchored in shared experience, (2) modeling successful personal recovery, and (3) mediating between perspectives of mental health service providers and service users (38).

To date, a number of peer training courses have been organized internationally, such as the certified Experienced Involvement (EX-IN) training program, which provides PSWs with the know-how to translate their lived experience into support for others. The program is a one-year qualification course based on a standardized curriculum and is designed to enable individuals with a history of mental health crises to work as “experiential experts” in established psychiatric care services (39). The training content was developed through a multidisciplinary and multilateral cooperation effort between six EU countries as part of the European Leonardo da Vinci pilot project EX-IN 2005–2007. The training consists of five basic and six advanced modules, which, drawing on individual experience and reflection thereupon, convey competencies on 11 key topics. The basic modules cover subjects such as “promoting health and well-being,” “empowerment in theory and practice,” “experience and participation,” “perspectives and experiences of recovery,” and “trialogue.” The advanced modules place greater emphasis on the practical aspects of working as a PSW. In addition, the program includes 40-h and 80-h internships, which are intended to transfer the theoretical content into everyday clinical practice (27).

Alongside the progress made in reconceptualizing recovery processes, a growing body of research has yielded important insights into the merits of peer support. Numerous studies demonstrate that peer support helps advance individual recovery (34, 40–42) by, e.g., having positive effects on patients’ empowerment (40–42), self-efficacy (42), feelings of hope (34, 42–44), and quality of life (44). Further beneficial effects include improved economic outcomes, such as a decrease in emergency service consultation (40, 45, 46) and enhanced cost-efficiency of mental health services (46). Moreover, peer support has been reported to enrich social rapport (41–43), contribute to the improvement of service provider/recipient relationships (40, 41), and heighten the recovery orientation of psychiatric facilities (28). Peer support also appears to be an asset not only to service users and institutions, but equally to PSWs, inducing a more profound experience of their personal recovery (42, 47).

Though several studies focusing on ways to avert events of restraint involved PSWs as part of more complex interventions (11, 48, 49), hitherto, scant attention has been paid to the effectiveness of peer support alone as a means of reducing restraint within acute psychiatric settings. There appears to be a discrepancy between the promotion of peer support and the absence of a body of literature—particularly from randomized controlled trials—devoted to investigating its efficacy (11, 28, 50). The present study intends to help close this research gap by modeling the effects of peer support on the use of restraint in adult patients in two equivalent mental health wards. It compared one locked acute ward where peer support was introduced in addition to routine care [*intervention group* (*IG*)] to a second locked acute ward in which treatment proceeded as usual, with no peer support provided [*control group* (*CG*)].

## 2. Methods

### 2.1. Peer-supported autonomy-promoting crisis treatment study

The data presented in this paper are part of the study “Peer-Supported Autonomy-Promoting Crisis Treatment” (PACT). The PACT study was conducted at the Department of Psychiatry, Psychotherapy and Psychosomatics at Vivantes Hospital Am Urban in Berlin, Germany, between 2018 and 2021. The PACT study assessed the impact of peer support on a broad range of patient-related and patient-reported outcomes, such as recovery-orientation, psychiatric symptoms, and service use. It was designed as a clinical, prospective, mixed methods controlled pre–post study and is registered at the German Clinical Trials Register (DRKS-ID: DRKS00015494).

### 2.2. Setting

Vivantes Hospital Am Urban treats patients within a catchment area of approximately 290,000 residents in Friedrichshain-Kreuzberg, an inner-city district of Berlin. It comprises one central emergency department, 11 departments of various medical specialties, and one psychiatric department. The psychiatric department houses 174 psychiatric inpatient beds and 50 day-clinic beds. It has two outpatient units. The hospital is situated in the center of a densely populated area of Berlin with grave social inequality and high drug abuse. This results in high admission rates of intoxicated and acutely ill people with resulting high rates of restraint. As a result, in both the intervention and the control ward participating in the present study, the Safewards model has been introduced in the time span between the years 2016–2018 (10). After the implementation of the Safewards model, the proportion of patients experiencing restraint in the two study wards numbered 23.0 and 5.6% (10). At the beginning of our study in 2018, the Safewards model was introduced on both wards with each of the ten Safewards interventions being fully implemented. None of the team members has worked on both wards at the same time. Moreover, neither has any other intervention with possible effects on rates of restraint or the ward climate been introduced, nor have any external factors been changed during the course of this study.

### 2.3. Intervention

Peer support was provided by two employees who were successfully trained as PSWs according to the EX-IN training program. The PSWs received a permanent contract of employment and were remunerated in accordance with the collective agreement for public service (TvöD). Each PSW worked around 7 h a week (27.5 h/month). Their introduction to the ward was aided by the senior management of the whole department. Immediately prior to the implementation of peer support, the wards’ health-care workforce organized a team building day aimed to reflect on the forthcoming introduction of PSWs, as well as to address potential hurdles and challenges. The lively discussions suggested that while the PSWs were awaited open-mindedly and with great curiosity, also uncertainty regarding the specific roles of the PSWs was expressed by the team. Consequently, the mentoring team drafted a workpace description referring to a specific manual (51).

For workplace support, PSWs were encouraged to take part in supervision: They were assisted by the senior psychologist of the ward who had previous experience with PSWs as well as four nurses who were especially assigned to mentor them. Supervision was provided biweekly by a minimum of two out of this group of five mentors and the senior consultant.

The PSWs engaged in a variety of activities and fields of responsibility. The three levels of engagement comprised: (1) initiating direct and personal contact with patients, (2) engaging in an exchange of experiences with traditional practitioners within the institutionalized health-care system, and (3) approaching decision-makers in order to facilitate and spur patient recovery and empowerment (51). During the course of the study, PSWs were found to have spent 60–70% of their working hours in direct contact with patients (of which 50–60% were exclusively spent with one patient, approximately 10% in group activities), 20% in exchange with other professional team members, and only less than 10% engaging with decision-makers, such as the senior nursing management or medical doctors. Beyond this, PSWs were granted some autonomy and flexibility to formulate their remit according to their own capacities and needs. PSWs engaged with patients in both individual (e.g., personal conversations) as well as group settings (e.g., joint leisure activities in the common room), and supported patients and staff alike in a variety of activities (e.g., offering patients company during their favored pastimes, training staff on recovery-focused matters, etc.). Training in recovery orientation was extended by the senior psychologist and the senior consultant of the ward in the form of on-the-job training. The principal themes comprised: People before diagnosis, patients’ specific psychological needs, socratic dialog, radical acceptance of patients’ preferences, radical validation, compiling recovery-oriented treatment plans, fostering social inclusion, empowerment, generating optimism and a positive sense of identity (29). Peer support took place in clinical as well as in outpatient settings (e.g., assisting staff in home treatment teams or joining patients in everyday activities in their private environment). PSWs furthermore worked either individually (e.g., peer counseling) or in collaboration with mental health professionals (e.g., co-moderation of open groups).

Unfortunately, we were unable to realize the schedule of the implementation as envisioned, which led to subsequent temporal adjustments. The two PSWs commenced their employment on the locked psychiatric ward at the same time (October 2018). However, unforeseen circumstances forced both PSWs to terminate their work arrangements prematurely during the course of implementation. Due to health reasons, the first PSW left the ward after 4 months (February 2019) and the second after 9 months (July 2019), resulting in a 5-month period of reduced peer support presence. One position was eventually refilled, 6 months after the second PSW dropped out (January 2020), while the second position remained vacant. Accordingly, several adjustments of the schedule were required in order to attain the planned total amount of working time: The intervention period with initially two and subsequently one PSW was extended until August 2020 with post-assessment conducted in September 2022, finally resulting in an implementation phase of 22 months instead of the planned 12 months. Furthermore, both assessments were extended to a period of 6 months, before and after the 22-month implementation process.

### 2.4. Treatment as usual and control condition

Standard inpatient care (treatment as usual, TAU) was offered in both the control and intervention groups. In the control condition no peer support was provided.

### 2.5. Restraint

The present study refers to the concept of restraint and its terminology by drawing on Negroni’s definitions elaborated in “On the Concept of Restraint in Psychiatry” (52). Accordingly, restraint can be defined as any measure applied that limits a patient’s personal freedom of movement. Furthermore, in the realms of psychiatry, restraint more concisely implies measures of a distinctly coercive nature. Negroni differentiates between “physical,” “chemical,” “environmental,” “psychological,” and “psycho-environmental” restraint. He further subdivides physical restraint into “manual,” “mechanical,” and “physical-psychological” restraint (52).

At Vivantes Hospital Am Urban, three types of restraint measures are employed in emergency situations: mechanical (physical) and chemical, as well as the combination thereof. Restraint in any form is strictly limited to situations which pose a critical threat to the patient’s or others’ well-being. Moreover, statutory regulations stipulate that the patient must demonstrate an inability to exercise self-determination before restraint may be considered (53). Mechanical restraint takes the form of fixation *via* wrist and ankle cuffs attached to the patient’s bed. Its application is strictly limited to situations in which no other means appear sufficient in order to prevent further harm (54). Chemical restraint describes the administration of medication without the patient’s consent. Medication may be dispensed orally or *via* intramuscular injection. For the purposes of standardization, events of chemical restraint were organized into two categories: chemical restraint applied alone (in which case the frequency of events of restraint was recorded) and chemical restraint applied together with mechanical restraint (in which case the total duration of the intervention was recorded) (52).

### 2.6. Data collection and outcomes

Two assessment periods of 3 months each—the first directly before the 12-month peer support implementation stage, the second directly after—were planned in order to collect data on the use of restraint. Both periods were extended from 3 to 6 months in order to more accurately document long-term effects and counterbalance variations in admission numbers caused by the 2019 coronavirus (COVID-19) pandemic. The implementation stage was itself extended to span the period from September 2018 to August 2020.

Data were gathered by one member of the research team who was not a member of the treatment team or otherwise involved in the implementation process. Generally, individuals obtain distinctive patient identification numbers (ID) when first admitted to the study hospital. The term *patient* refers to this ID, which is retained for subsequent admissions. Routine basic documentation includes medical (main diagnosis) and sociodemographic data (migration background according to nationality, sex, and age), as well as information regarding allocation to IG or CG. Patients were assigned to diagnostic blocks based on chapter V of the ICD-10 manual (55). Data on nationality, sex, and age were documented at admission. Data on events of restraint were drawn from manual records kept by the hospital administration, which is a standardized procedure. These data include the restraint technique applied, the starting and end point of the measure, as well as the duration of restraint. Patients who were treated both in IG and CG during post-assessment were excluded from further data analysis.

Medical and sociodemographic information was collected for all patients treated during the assessment periods in the study wards (IG as well as CG) to compare these two groups. The main analyses of this study focus on restraint-related data for those patients in IG and CG who were exposed to restraint. We assessed the total number of events of restraint (all three types of restraint), and the duration of mechanical restraint (with or without forced medication) in total and per patient (56–59). The outcome indicators, which were adopted in accordance to those accredited by the German Working Group for the Prevention of Violence and Coercion in Psychiatry and reported separately for each method of restraint, were as follows: percentage of cases subjected to restraint (of all cases treated), mean duration of restraint, and duration and number of events of restraint per case (60, 61). Steinert et al. voiced concerns about possible violations of the Data Protection Act when analyzing data from several hospitals with reference to individual patients (62). However, the data of the present study are limited to patients of only one hospital. Thus, outcome indicators were adjusted to patient-wise analyses.

### 2.7. Statistical analysis

The evaluation was conducted based on a statistical plan that was elaborated prior to the implementation of peer support. Data analysis was performed using IBM SPSS Statistics 28. Valid data on the application of restraint in acute psychiatric wards in Germany are scarce. We therefore refrained from any preceding power analysis. The data were analyzed with reference to two levels of observation which were consistent with different reference subsamples. The samples were composed of and will be referred to as (1) total number of *events of restraint* and (2) total number of *patients* subjected to different types of restraint.

Continuous variables were tested for normal distribution by means of Q–Q plots, histograms, and Kolmogorov–Smirnov tests. Based on the results, continuous variables were presented as mean [standard deviation (SD)] or median (25th; 75th percentile). In addition, the range was reported. Nominal variables were presented as absolute (*n*) and relative frequencies (*%*).

Chi-square tests were performed to measure group differences with regard to nominally scaled variables. If normal distribution was determined, group and pre–post comparisons of continuous variables were assessed by an unpaired *t*-test; if normal distribution did not occur, a Mann–Whitney *U* test was performed. Statistical significance was determined by a *value of p* < 0.05. The effect size (*r*) was reported for the Mann–Whitney *U* test and unpaired *t*-test. Phi, or Cramer’s V, was reported for Chi-square tests. For all effect sizes reported here, 0.1 indicates a small, 0.3 a moderate, and 0.5 a strong statistical effect. With regard to the duration of restraint, the effect size was reported along with its 95% confidence interval (CI). Furthermore, a multiple linear regression was undertaken with regard to all patients treated during post-assessment in order to assess predictors for the number of events of restraint that patients were subjected to. Beta ± 1 standard error (SE) and its 95% CI, as well as *value of p*, were presented for each predictor. The corrected *R*^2^ and the model summary statistics were given as indicators of the model’s overall quality.

## 3. Results

Prior to the statistical analyses, 24 patients who were treated both in IG and CG (i.e., who had treatment episodes on both wards) during the post-intervention assessment period were excluded from further data analyses, finally resulting in a sample that consisted of 923 patients (*n*_pre t0_ = 484; *n*_post t1_ = 439; see Table 1).

**Table 1:** Sociodemographic and disease-related data per group (IG or CG) with regard to *all patients treated* before (pre) and after (post) the implementation of peer support (*n* = 923).

Variable	Category	Intervention group *n* = 484			Control group *n* = 439			Intervention vs. control group value of *p*, effect size	
		Pre-assessment *n* = 284	Post-assessment *n* = 200	Value of *p*, effect size	Pre-assessment *n* = 266	Post-assessment *n* = 173	Value of *p*, effect size	Pre-assessment *n* = 550	Post-assessment *n* = 373
Sex, *n (%)*	Male	165 (58.1%)	124 (62.0%)	0.389^1^, −0.039^2^	162 (60.9%)	113 (65.3%)	0.350^1^, −0.045^2^	0.503^1^, 0.029^2^	0.507^1^, 0.034^2^
Migration background, *n (%)*	No	280 (98.6%)	167 (83.5%)	**<0.001**^1^, 0.280^2^	255 (95.9%)	147 (85.0%)	**<0.001**^1^, 0.192^2^	**0.050**^1^, −0.084^2^	0.698^1^, 0.020^2^
Diagnosis group, *n (%)*	F01 Organic, including symptomatic, mental disorders	6 (2.1%)	6 (3.0%)		5 (1.9%)	2 (1.2%)			
	F1X Mental and behavioral disorders due to psychoactive substance use	87 (30.6%)	75 (37.5%)	0.115^1^, 0.072^2^	71 (26.7%)	67 (38.7%)	**0.008**^1^, 0.127^2^	0.307^1^, 0.044^2^	0.808^1^, −0.013^2^
	F2X Schizophrenia, schizotypal, and delusional disorders	103 (36.3%)	81 (40.5%)	0.345^1^, 0.043^2^	124 (46.6%)	71 (41.0%)	0.251^1^, −0.055^2^	**0.014**^1^, −0.105^2^	0.916^1^, −0.005^2^
	F3X Affective disorders	39 (13.7%)	13 (6.5%)	**0.011**^1^, −0.115^2^	31 (11.7%)	16 (9.2%)	0.426^1^, −0.038^2^	0.465^1^, 0.031^2^	0.323^1^, −0.051^2^
	F4X Neurotic, stress-related, and somatoform disorders	22 (7.7%)	7 (3.5%)		18 (6.8%)	6 (3.5%)			
	F5X Behavioral syndromes associated with physiological disturbances and physical factors	1 (0.4%)	–		–	–			
	F6X Disorders of adult personality and behavior	22 (7.7%)	10 (5.0%)		14 (5.3%)	10 (5.8%)			
	F7X Mental retardation	–	1 (0.5%)		1 (0.4%)	–			
	F8X Pervasive and specific developmental disorders	–	1 (0.5%)		1 (0.4%)	–			
	Non-psychiatric main disorder	4 (1.4)	6 (3.0)		1 (0.4%)	1 (0.6%)			
Age, mean (SD) [range]		40.29 (15.38) [18–108]	39.01 (14.96) [17–100]	0.367^3^, 0.062^4^	39.17 (14.08) [18–98]	38.56 (12.47) [18–85]	0.644^3^, 0.046^4^	0.377^3^, 0.057^4^	0.750^3^, 0.041^4^

^1^Value of *p* according to *χ*^2^ test. ^2^Effect sizes according to phi or *V*. ^3^Value of *p* according to two-sided *t*-test for independent samples. ^4^Effect sizes for *t*-test. Interpretation of the effect sizes: 0.1: small effect, 0.3: medium effect, 0.5: large effect. bold=statistically significant.

### 3.1. Sociodemographic outcomes and comparability of study groups

For statistical analyses, the most frequent main diagnoses consistent across groups and study periods were selected (FX1, FX2, and FX3). Sociodemographic data for all patients treated are reported in Table 1, showing that during pre-assessment the examined study cohorts did not differ significantly with regard to age and sex, and diagnosis groups F1X and F3X, whereas a significant difference was observed for migration background (variable label “No,” pre: *n*_CG_ = 255 [95.9%] vs. *n*_IG_ = 280 [98.6%], [*p* = 0.050, *phi* = −0.084]) and diagnostic group F2X (pre: *n*_CG_ = 124 [46.6%] vs. *n*_IG_ = 103 [36.3%], [*p* = 0.014, *phi* = −0.105]). During post-assessment, no statistically significant differences were observed between IG and CG.

### 3.2. Risk factors for frequency of restraint

After the implementation of peer support (post-assessment), neither female sex (beta = 0.37 ± 0.25, 95% CI [−0.13; 0.87], *p* = 0.151), migration background (beta = −0.45 ± 0.33, 95% CI [−1.10; 0.21], *p* = 0.179), age (beta = −0.01 ± 0.01, 95% CI [−0.02; 0.01], *p* = 0.441), nor allocation to IG (beta = 0.08 ± 0.24, 95% CI [−0.40; 0.56], *p* = 0.737) influenced the number of measures of restraint patients were subjected to.

### 3.3. Frequency of restraint

Overall, restraint was administered on 479 occasions over both study periods. Mechanical restraint occurred most frequently (*n* = 438 [91.4%]), followed by the combination of mechanical restraint and forced medication (*n* = 38 [7.9%]). Forced medication alone was administered infrequently (*n* = 3 [0.6%]) and will thus not be considered for further statistical comparison.

Within CG, the proportion of patients experiencing restraint significantly increased from pre- to post-assessment (CG_pre_: 44/266 [16.5%] vs. CG_post_: 46/173 [26.6%], *p* = 0.011, *phi* = 0.122), while there was no change in the intervention group (IG_pre_: 72/284 [25.4%] vs. IG_post_: 43/200 [21.5%], *p* = 0.327, *phi* = −0.045; see Table 2).

**Table 2:** Restraint-related data per group (IG or CG) regarding *all patients treated* before (pre) and after (post) the implementation of peer support (*n* = 923).

Variable	Category	Intervention group *n* = 484			Control group *n* = 439		
		Pre-assessment *n* = 284	Post-assessment *n* = 200	Value of *p*, effect size	Pre-assessment *n* = 266	Post-assessment *n* = 173	Value of *p*, effect size
Restraint	Yes	72 (25.0%)	43 (21.5%)	0.327^1^, −0.045^2^	44 (16.5%)	46 (26.6%)	**0.011**^1^, −0.122^2^
Frequency of restraint per patient (Median [25th; 75th percentile])		1 [1; 2]	1 [1; 2]	0.153^3^, −0.126^4^	1 [1; 1]	1 [1; 3]	**0.018**^3^, −0.242^4^

Value of *p* according to the *χ*^2^ test. ^2^Effect sizes according to *phi*. ^3^Value of *p* according to two-sided Mann–Whitney *U* test for independent samples. ^4^Effect sizes according to *r*. Interpretation of the effect sizes: 0.1: small effect, 0.3: medium effect, 0.5: large effect. bold=statistically significant.

Similarly, the frequency of restraint per patient throughout the hospital stay increased significantly in CG from pre- to post-assessment (CG_pre_: 1 [1; 1] vs. CG_post_: 1 [1; 3], *p* = 0.018, *r* = −0.242), while no change was observed in IG (IG_pre_: 1 [1; 2] vs. IG_post_: 1 [1; 2], *p* = 0.153, *r* = −0.126).

**Figure 1:**
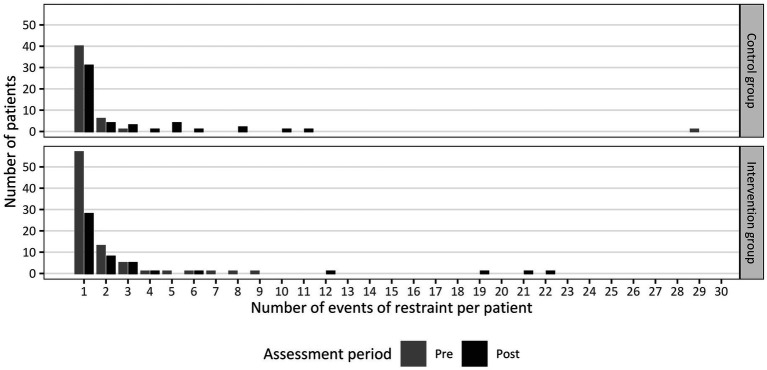
Number of events of restraint *per patient* separated by group (Intervention Group or Control Group) before (pre) and after (post) the implementation of peer support (*n*_t0_ = 116, *n*_t1_ = 103).

It is important to note that due to its strongly skewed distribution, the median [(25th; 75th) percentile] are reported for the frequency of restraint per patient and duration of restraint instead of the mean and standard deviation as originally suggested by Steinert et al. (60, 61). See Figure 1 for a graphic summary of the range and distribution of frequencies of restraint of both groups.

### 3.4. Duration of restraint

With regard to the total number of events of restraint, no change in the duration of restraint was observed across study periods, irrespective of study group (CG: *p* = 0.068, *r* = −0.130; IG: *p* = 0.502, *r* = −0.040, details presented in Table 3).

**Table 3:** Duration (in hours) of events of restraint with regard to *the total number of events of restraint* separated by group (IG or CG) before (pre) and after (post) the implementation of peer support (*n* = 476).

Variable	Pre-assessment			Post-assessment			Value of *p*	Effect size^1^	95% CI^3^
	*n*	Med^2^ [25th; 75th Percentile]	Range	*n*	Med^2^ [25th; 75th Percentile]	Range			
**Mechanical restraint**	191	2.17 [1.00; 4.43]	0.08–126.83	247	2.32 [0.92; 5.50]	0.25–30.15	0.665	−0.021	−0.115, 0.073
Intervention group	116	2.75 [1.44; 6.35]	0.08–126.83	134	3.00 [1.08; 6.21]	0.33–30.15	0.746	−0.020	−0.144, 0.104
Control group	75	1.50 [0.67; 2.42]	0.08–16.00	113	2.00 [0.71; 4.33]	0.25–29.83	0.122	−0.114	−0.253, 0.030
**Combination: Mechanical restraint and forced medication**	27	2.25 [1.08; 6.33]	0.08–34.25	11	3.33 [0.50; 9.00]	0.17–11.00	0.974	−0.005	−0.324, 0.315
Intervention group	19	6.00 [1.67; 9.70]	0.08–34.25	9	3.33 [1.00; 9.08]	0.50–11.00	0.498	−0.130	−0.480, 0.256
Control group	8	1.21 [0.44; 1.48]	0.42–3.50	2	3.17 [0.17; −]	0.17–6.17			
**Restraint overall**	218	2.21 [1.06; 5.00]	0.08–126.83	258	2.33 [0.92; 5.63]	0.17–30.15	0.752	−0.014	−0.104, 0.076
Intervention group	135	2.83 [1.50; 6.50]	0.08–126.83	143	3.17 [1.08; 6.33]	0.33–30.15	0.502	−0.040	−0.157, 0.078
Control group	83	1.42 [0.67; 2.42]	0.08–16.00	115	2.00 [0.67; 4.33]	0.17–29.83	0.068	−0.130	−0.265, 0.010

^1^Effect sizes for Mann–Whitney *U* test. Interpretation of the effect sizes: 0.1: small effect, 0.3: medium effect, 0.5: large effect. ^2^Med = Median. ^3^CI = Confidence Interval.

Similar results were obtained with regard to individual patients who were subjected to restraint: no significant change in the duration of restraint was found for either CG (*p* = 0.069, *r* = −0.187) or IG (*p* = 0.265, *r* = −0.099, see Table 4).

**Table 4:** Duration (in hours) of events of restraint with regard to *patients who were exposed to restraint* separated by group (IG or CG) before (pre) and after (post) the implementation of peer support (*n* = 222)^1^.

Variable	Pre-assessment			Post-assessment			*p*-Value	Effect size^2^	95% CI^4^
	n	Med^3^ [25th; 75th Percentile]	Range	n	Med^3^ [25th; 75th Percentile]	Range			
**Mechanical restraint**	103	2.42 [0.87; 4.48]	0.08–61.25	86	2.98 [1.49; 5.48]	0.25–26.00	0.235	−0.086	−0.226, 0.057
Intervention group	63	2.83 [1.78; 6.67]	0.08–61.25	40	4.21 [2.83; 6.50]	0.42–26.00	0.159	−0.139	−0.324, 0.056
Control group	40	1.25 [0.52; 2.57]	0.08–16.00	46	1.83 [0.81; 4.42]	0.25–20.00	0.121	−0.167	−0.366, 0.047
**Combination: Mechanical restraint and forced medication**	24	2.29 [1.13; 8.27]	0.08–34.25	9	4.00 [2.33; 7.58]	0.17–9.17	0.736	−0.063	−0.398, 0.287
Intervention group	17	6.25 [1.83; 10.35]	0.08–34.25	7	4.00 [3.17; 9.00]	1.50–9.17	0.757	−0.071	−0.461, 0.342
Control group	7	1.25 [0.42; 1.53]	0.42–2.33	2	3.17 [0.17; −]	0.17–6.17			
**Restraint overall**	127	2.33 [1.00; 5.83]	0.08–61.25	95	3.17 [1.50; 5.50]	0.17–26.00	0.233	−0.080	−0.210, 0.052
Intervention group	80	3.06 [1.81; 6.98]	0.08–61.25	47	4.00 [2.93; 6.50]	0.42–26.00	0.265	−0.099	−0.269, 0.077
Control group	47	1.25 [0.50; 2.33]	0.08–16.00	48	1.83 [0.60; 4.58]	0.17–20.00	0.069	−0.187	−0.374, 0.015

^1^Since patients were seldom subjected to several forms of restraint, the analyzed number of patients displayed in this table negligibly exceeds the total number of patients subjected to restraint. ^2^Effect sizes for Mann–Whitney *U* test. Interpretation of the effect sizes: 0.1: small effect, 0.3: medium effect, 0.5: large effect. ^3^Med = Median. ^4^CI = Confidence interval.

## 4. Discussion

To the best of our knowledge, the present study was the first to set out to explore the relationship between peer support and the use of restraint in acute psychiatry and on locked wards more specifically. In the observation period after the implementation of peer support in the intervention group, both groups were comparable with respect to all characteristics considered. We could show that with no peer support available in the control group, the proportion of patients subjected to restraint as well as of the frequency of restraint per patient significantly increased across study periods, whereas in the intervention group, no change was seen. The results further indicate no intervention effects for the duration of restraint in either of the study groups. Contrary to our expectations, peer support therefore was not associated with a reduction in the frequency of restraint and the duration of these measures.

These findings on the possible effects of peer support on the use of restraint unfortunately cannot be juxtaposed with any previous results, as no evidence from controlled studies on this matter has yet been established (11). Considering the vast variability in the use of restraint across countries, it appears reasonable to focus on relating additional findings of our study to those from other German hospitals. Compared with the percentage of patients subjected to restraint reported in a study by Adorjan et al. (8%) (20), the values in the present study were considerably higher (16.5–26.6%). A possible reason for this discrepancy might be that the sample of Adorjan et al. consisted of heterogenous hospitals and psychiatric wards, while we exclusively analyzed data from locked wards in Vivantes Hospital Am Urban. The prevalence of restraint explicitly reported for acute psychiatric wards in German hospitals could not be derived from existing literature. Nevertheless, the results of our analysis coincide with those of Adorjan et al. in terms of the number of events of restraint per patient; further findings from the same study correspond with our finding of mechanical restraint being the most commonly used form of restraint in Germany (20).

Besides the significant increase in both the proportion of patients subjected to restraint and the frequency of restraint per patient observed for the control group, on a descriptive level, we identified a rather indeterminate upward trend in the duration and total number of events of restraint in both groups that exceeded seasonal fluctuations. Although this increase remained below the threshold of statistical significance, it draws attention to a more general, yet unspecified, effect impacting on both wards during the course of our study.

Declared an international public health emergency on January 30, 2020 by the World Health Organization (WHO) (63), COVID-19 has profoundly affected our clinical practice and rendered clinical processes noticeably challenging, particularly during the post-assessment period. This, however, applies to both wards in the same way. A plethora of studies have since investigated the impact of COVID-19 on mental health. The general picture emerging from these analyses is that mental well-being has deteriorated significantly with the outbreak of the virus, in patients as well as health-care professionals. The pandemic’s detrimental effects, which we see reflected in our own everyday experiences, comprise, e.g., increasing levels of mental distress (64, 65) and symptomatology (66, 67), as well as a possible surge in episodes of mental disorders (68, 69). Clinical experience shows that elevated symptom severity adversely impacts the duration and frequency of interaction between peer support workers and patients. Furthermore, patients with higher scores of symptom severity are more likely to be subjected to restraint (70–72). In addition, sick leaves of health-care providers on direct or indirect account of COVID-19, e.g., due to the psychological repercussions of the pandemic, have led to a substantial problem of understaffed wards in our clinics, inducing even more work-related distress. Another aggravating factor that we ascertained was striking workforces, which presumably contributed to feelings of uncertainty in both personnel and patients. These circumstances have precipitated an imbalance in the patient–staff ratio that may have resulted in a general increase in the use of restraint. Therefore, in the face of the pandemic and its extensive effects on mental health and medical care, it can be tentatively hypothesized that peer support may have had a protective effect with regard to the duration of events and the percentage of patients subjected to restraint as well as the frequency of restraint per patient. However, such inferences must be drawn with caution in the absence of a closer look at causal and contributory factors for the observed general surge in restraint, such as the ongoing pandemic situation.

Another factor that had a possible impact on our study may have been the change of the consulting psychiatrist in the intervention ward during the implementation period. Moreover, sick leaves may also have limited the effect of PSWs on the team and the processes on the intervention ward. Longer absences of peers led to discontinuities that we may have only partly compensated for with the extension of the intervention and assessment periods. Both aforementioned factors may have prevented peer support from having a greater impact. Overall, peer support is a complex, person-centered intervention susceptible to interferences on many levels and with wide-ranging effects on different aspects of the clinical and organizational setting. Although the implementation of peer support was carefully prepared and eagerly supported by senior management, it did not appear achievable to provide for every eventuality. It was noted earlier that it seemed prudent to grant PSWs some autonomy in order to extend their flexibility to respond to personal needs. By implication, we accepted some uncertainty with regard to, e.g., PSWs’ time spent in direct contact with patients or staff. This decision may have limited PSWs’ anticipated positive impact on restraint in the pre–post comparison.

### 4.1. Limitations

In interpreting the findings of our study, several limitations must be considered. This was not a randomized study, as individual randomization did not seem feasible—a problem well known in this field of research (13). However, although both the intervention as well as the control group were comparable with respect to all of the characteristics considered during post-assessment, we cannot rule out the possibility of differences in relevant areas of comparison that we have not included in our analysis and that may have had an impact on the comparative prevalence of restraint. Additional factors that may be pertinent to decision-making with regard to restraint may include staff and organizational or clinical matters such as—as noted earlier—severity of symptoms (70). Another limitation to this study has been reiterated by numerous researchers before, namely, the substantial heterogeneity of peer work and its outcomes (73, 74), as well as implementation processes (75). Utilizing existing fidelity indexes (76, 77), implementation frameworks (78), or guidelines (33) may facilitate further inquiry into the relationship between peer support and restraint.

As both PSWs discontinued their work during the course of the study, we subsequently had to refill the positions adequately resulting in an interruption of the intervention. This unforeseen disturbance challenges the replicability of our study. However, we believe that by adjusting each study period according to the planned total amount of peer support working hours and correspondingly granting the effects of the intervention additional time to unfold, we were able to partly compensate for the inconvenient disruptions.

### 4.2. Conclusion

This is a first and preliminary study that sought to empirically substantiate the claim of the S3 guideline “Prevention of Coercion: Prevention and Therapy of Aggressive Behavior in Adults” that the presence of PSWs in psychiatric hospitals and potentially other settings may be an effective measure to reduce the use of restraint. We conclude that—compared to treatment as usual—the intervention may have had some protective effect on patients treated in an acute psychiatric environment. While in the control group the application of restraint significantly increased with regard to the proportion of patients subjected to restraint (of all patients treated) and the frequency of restraint per patient, in the intervention group no statistically significant change was observed. These findings justify and encourage future research to investigate the full preventive potential of incorporating PSWs in the mental health-care workforce. The challenge for future research will be to control other independent factors with a possible impact on conflicts and restraint measures in acute psychiatric wards in order to identify the mechanisms underpinning peer support in mental health institutions.

## Data availability statement

The original contributions presented in the study are included in the article/Supplementary material, further inquiries can be directed to the corresponding author.

## Ethics statement

This study is registered at the German Clinical Trials Register (DRKS-ID: DRKS00015494).

The studies involving human participants were reviewed and approved by Medical Ethics Committee of Charité University Hospital (ethical approval number EA2/137/18). The patients/participants provided their written informed consent to participate in this study.

## Author contributions

SW and JoB designed the study. JoB closely supervised the implementation and evaluation process of the study. JuB collected, analyzed, and interpreted the data. JuB furthermore designed the outline of the manuscript and drafted the first version of the manuscript. All authors critically revised the said manuscript. All authors contributed to the article and approved the submitted version.

## Conflict of interest

The authors declare that the research was conducted in the absence of any commercial or financial relationships that could be construed as a potential conflict of interest.

The handling editor MS declared a shared affiliation with the author AB at the time of review.

## Publisher’s note

All claims expressed in this article are solely those of the authors and do not necessarily represent those of their affiliated organizations, or those of the publisher, the editors and the reviewers. Any product that may be evaluated in this article, or claim that may be made by its manufacturer, is not guaranteed or endorsed by the publisher.
